# “This is really something: same place, same day result, same day treatment” women’s experiences of testing positive for HPV and receiving same-day treatment in Papua New Guinea: an interpretative phenomenological analysis

**DOI:** 10.1186/s12905-023-02557-z

**Published:** 2023-08-18

**Authors:** Hawa Camara, Somu Nosi, Gloria Munnull, Steven G Badman, John Bolgna, Joseph Kuk, Glen DL Mola, Rebecca Guy, Andrew J. Vallely, Angela Kelly-Hanku

**Affiliations:** 1 Kirby Institute for Infection and Immunity in Society, UNSW Sydney, Wallace Wurth Building, UNSW, Sydney, NSW 2052 Australia; 2 Papua New Guinea Institute of Medical Research, Homate Street, PO Box 60, Goroka, Eastern Highlands Province Papua New Guinea; 3 Department of Obstetrics & Gynaecology, Mt Hagen Provincial Hospital, WHP 281, PO Box 36, Mt Hagen, Papua New Guinea; 4 Department of Obstetrics & Gynaecology, Modilon General Hospital, PO Box 1200, Madang, Papua New Guinea; 5 School of Medicine and Health Sciences, University of Papua New Guinea, NCD, PO Box 5623, Boroko, Papua New Guinea

**Keywords:** HPV positive test result, Same day screen-and-treat, Cervical screening, Papua New Guinea, Thermal ablation

## Abstract

**Introduction:**

Human papillomavirus (HPV) testing is transforming cervical screening globally. The World Health Organization (WHO) now recommends same-day HPV screen-and-treat for primary cervical screening in low- and middle-income countries (LMIC) but there is a lack of evidence on women’s lived experience of testing positive for oncogenic HPV and receiving same-day treatment. This study aimed to address this knowledge gap among women participating in a same-day HPV screen-and-treat (HPV S&T) program in Papua New Guinea.

**Methods:**

As part of a larger qualitative study, this paper explores the lived experiences of 26 women who tested positive for oncogenic HPV and were treated the same day. We analysed the data using the interpretative phenomenological analysis method. All data were managed using Nvivo 12.5.

**Results:**

The interpretative phenomenological analysis led to three superordinate themes: 1) facing and alleviating initial worries, (2) transforming the disclosure process, and (3) connecting to their faith. Women’s experiences of the same day HPV screen-and-treat were framed by initial emotional reactions to their positive HPV test result, and having access to treatment on the same day, which helped address their worries and fears, and transformed their experience of disclosing their test result and subsequent treatment to family and friends.

**Conclusion:**

This study shows that, while women experience similar initial emotional reactions, undergoing same day treatment quickly resolved the women’s worries, making this program highly acceptable. Overall, women’s engagement in the program confirmed its high acceptability and cultural congruence, leaving women feeling empowered and hopeful about their future, and the future of all Papua New Guinea women.

## Introduction

Cervical cancer is a leading cancer affecting women and individuals with a cervix globally, with close to 85% of cases occurring in low- and-middle-income countries (LMIC) [1, 2]. Research has shown that persistent infection with oncogenic strains of the sexually transmitted infection, the human papillomavirus (HPV), causes cervical cancer. There are 14 oncogenic HPV types known to cause over 99% of cervical precancer and cancer cases, with HPV 16 and 18 being responsible for 70% of all cases [3–5]. Recent technological innovations include high-performing and cost-effective HPV-based molecular platforms and that accurately detect oncogenic HPV types [6–8], some of which can be used at point-of-care, such as the GeneXpert platform (Cepheid, Sunnyvale, CA), to provide women with same-day test results [9–11]. In 2021, the World Health Organization (WHO) recommended same-day (or single visit) HPV screening and precancer treatment for primary cervical screening in low- and middle-income countries [8, 12, 13]. In addition to point-of-care diagnostics, thermal ablation is recommended for same-day treatment of cervical precancer due to its superior clinical efficacy and adverse events profile compared with earlier technologies such as cryotherapy [13, 14]. The advances in cervical screening from the Papanicolaou test (Pap test) to new molecular point-of-care platforms that can detect HPV and with same day treatment possible with thermal ablation has revolutionised the biomedical landscape of screening (and treatment), most notably for women in low- and middle-income countries.

Reflective of the historical approach to cervical screening, much of the social research on diagnosis and illness narratives to date has focused on experiences of receiving abnormal Pap test or histology results and of receiving a diagnosis such as cervical precancer or cancer [15–17]. With the identification that HPV is the primary cause of cervical cancer, the diagnosis of cervical (pre)cancer was transformed to a cancer imbued with stigma due to the virus being a sexually transmitted infection (STI). The shift in screening practices to be tested for a cancer-causing STI has significant impacts on the ways in which women now make sense of testing positive, bringing to the forefront personal and interpersonal meanings of a positive HPV test result.

While the recent introduction of HPV testing affords a level of certainty that is absent from traditional cervical screening methods (i.e., Pap tests and visual inspection with acetic acid, VIA), that specificity leads to new issues for women who test positive for HPV. As HPV testing expanded, so has the social research which examines the cervical cancer-related illness narratives of women testing positive for HPV. A recent global systematic review and meta-analysis synthesised evidence from Australia, Austria, Brazil, Canada, Greece, Italy, Ireland, Mexico, Norway, Tanzania, Turkey, the UK, and the USA on emotional responses associated with a positive HPV test result [18]. It found that most women experienced varied levels of anxiety, as well as emotions such as hopelessness, sadness, shame, and blame (self and toward partner), to name a few. These emotional responses mainly stemmed from the fear of developing cervical cancer, the stigmatized connection to an STI and its subsequent impact on their social identity [19–24]. Most societies associate the detection of HPV with a lack of hygiene, promiscuity, and immorality [25–29]. This is particularly salient in societies that shame women who engage in premarital sexual activity [30, 31]. Stigma and shame have also impacted other aspects of the women’s screening experience, such as the disclosure process. Women end up dreading the disclosure of a positive test result for fear of being shunned by key members of their social circles [32, 33].

The existing evidence only pertains to women who have undergone screening services in the absence of same day treatment. With the expansion of screening and treatment technologies in cervical screening, we cannot help but wonder how women who test positive for HPV and are subsequently treated on the same day make sense of their diagnosis and their emotional responses to it, and how this affects their sense of self. In this paper we use an interpretative phenomenological analysis to explore the experiences of women testing positive for HPV and undergoing same day treatment in a novel same day screen-and-treat program conducted in Papua New Guinea (2018–2021). This is certainly the first paper from the Pacific that examines women’s illness narratives as part of a point-of-care self-collect HPV screen-and-treat program, and to our knowledge the first globally, and as such offers an important opportunity to examine how same day treatment moderates women’s illness narratives.

## Methods

This qualitative study is part of a large-scale field trial conducted in Papua New Guinea which aimed to evaluate the clinical performance, cost-effectiveness, acceptability, and health systems requirements of the self-collect HPV screen-and-treat program to inform future national and global programs [5, 10, 11, 34]. We designed a qualitative study exploring women’s, health care workers, and policymakers’ experiences of HPV S&T using self-collection at the two trial sites, Modilon General Hospital in Madang Province, and Mount Hagen General Hospital in Western Highlands Province. Elsewhere, we have examined women’s acceptability of HPV S&T [35], health care workers and key informants’ perspectives of HPV S&T [36], and in this paper we use an interpretative phenomenological analysis to explore the experiences of women who tested positive for HPV and were treated on the same day.

### Setting

Papua New Guinea (PNG) is a culturally diverse country in the Pacific with high rates of cervical cancer incidence and mortality [37, 38]. Previous cervical screening programs yielded poor performances and health outcomes, screening less than 5% of age-eligible women nationally [34, 39]. Currently, cervical cancer screening and treatment services are infrequent, ineffective and mostly inaccessible [10, 39].

In 2018, a field trial was initiated to evaluate a same-day point of care HPV self-collect screen-and-treat program (HPV S&T) in Papua New Guinea. In the PNG trial, women who were tested positive for HPV underwent same-day visual examination of the cervix to assess eligibility for treatment and if eligible, were offered same-day treatment of the cervical by thermal ablation [6]. A clinical review was scheduled three months after their treatment to confirm post-treatment resolution. Women who were ineligible for same day treatment (e.g., because a lesion suspicious of cervical cancer was seen on examination) were referred for gynaecological review at their local provincial hospital.

### Sampling

Health care workers employed by the Papua New Guinea Institute of Medical Research (PNGIMR) to work at the Well Woman Clinics’ (WWC) and lead the HPV S&T program purposively identified a heterogeneous sample of 62 women who had participated in the program, and who could provide diverse and information-rich data to help address our research questions [40]. To examine the experiences of testing positive for HPV and undergoing same day treatment we draw on data from a sub-sample of 26 women who self-reported that they had tested positive for HPV. All but one of the 26 shared that they had been provided same day thermal ablation treatment. Upon visual inspection one woman was identified by the treating nurse as being ineligible for thermal ablation and instead was referred for further gynaecological examination (i.e., biopsy).

### Data collection

A semi-structured interview guide was developed and used to interview all women. We used semi-structured interviews due to the sensitive and intimate nature of the questions and to provide a comfortable environment to discuss descriptive details of the women’s experiences [40]. As part of the overall interview guide, we had a subset of questions that were specifically designed to only be asked of the women who self-reported having tested positive for HPV. In these interviews, we enquired about the meaning of their test result and their experience of treatment, as well as aspects associated with disclosure of their test result.

Information and consent forms explaining the purpose of the study were provided ahead of the interview in a language of their choosing (available in both English and Tok Pisin). All participant details are deidentified and names used in this paper are pseudonyms. The interviews were conducted between May 2018 and August 2019 and took place either on the day of their screen-and-treat procedure or within a few days after, reducing the risk of recall bias. It also meant women’s responses were relatively immediate. We acknowledge that illness narratives can and do change over time [41], but as these women were provided same day treatment, it was important that we capture their experience of testing positive and receiving treatment as close to the event as possible. We interviewed women from diverse geographic regions including women from remote areas who had travelled several days to participate in the program.

Interviews took place in a private office space at the clinic and/or in private spaces outside the clinic (at a hotel/lodge where the participants were residing or the interviewers) to ensure confidentiality and privacy of participants. Interviews were audio-recorded and conducted either in English, Pidgin (Tok Pisin) or a mix of both English and Tok Pisin. Interviews averaged 40 min but ranged from half an hour to just over an hour. All interviews were audio-recorded and transcribed verbatim and then translated into English as necessary. All interviews transcribed in Pidgin and then translated into English were led by a team of highly experienced staff at the Papua New Guinea Institute of Medical Research (PNGIMR).

### Data Analysis

The data were analysed using the interpretative phenomenological analysis method, an idiographic (i.e., a person making sense of their lived experience) and hermeneutics (i.e., theory of interpretation), which focuses on the lived experience of individuals [42]. As this study aimed to explore the lived experience of women who had tested positive for HPV and were treated on the same day, an interpretative phenomenological analysis methodology was deemed appropriate because it is used and designed to examine the existential experience of a unique and homogeneous group of individuals [42–44]. An interpretative phenomenological approach privileges how people make sense of their personal and social world, by focusing on personal experiences and perceptions of a specific phenomenon or event [43]. An Interpretative phenomenological method uses a dynamic and systematic approach to examine how a particular phenomenon has been understood from the perspective of particular people (emic), in a particular context [44, 45].

The interpretative phenomenological approach differs from the Thematic Analysis (TA) method in that its duality (subjective and idiographic nature) requires for the methodology to specifically focus on participants’ unique individual characteristics and unique subjective lived experience. Meanwhile, the thematic analysis is specific to only identifying patterns across all participants. Analytically, the coding process is also dissimilar as TA proceeds with data familiarization, and coding across the entire data set.

The interpretative phenomenological analysis process requires immersion into the data to understand the meanings being generated by each participant [43, 44]. Each transcript was read numerous times and analysed separately to identify key codes. A codebook was developed accordingly by the first author and used to identify emerging themes by finding patterns across the codes. These emerging themes describe key elements of the women’s lived experiences. The author provided quotes to illustrate the themes presented in the findings. All data were managed using NVivo 12.5 (QSR International, ltd.) [46].

### Ethical considerations

The field trial and sub-studies was approved by the Papua New Guinea Institute of Medical Research (PNGIMR) Institutional Review Board (IRB) (approved in November 2017, No. 1712), the Medical Research Advisory Committee (MRAC) of the PNG National Department of Health (NdoH) (approved in September 2017, No. 17.36), and the University of New South Wales (UNSW) Human Research Ethics Committee (approved in September 2017, No. HC17631). All methods were carried out in accordance with relevant guidelines and regulations.

## Findings

### Participant characteristics

Of the 26 women who reported that they tested positive for HPV, 22 were between 30 and 49 years of age, the average age being 40 years. The majority of the women (n = 19) were in a relationship or married. Most of the women (n = 23) had one or more children, and 15 of the women were formally employed. Half of the women had an education level up to Grade 12. Thirteen of the 26 HPV positive women interviewed in this study were residing in Madang Province, 10 lived in Western Highland Province, two in Southern Highlands Province, and one in Simbu Province. Women who identified as Christian included a range of religious denominations: Lutheran (n = 5), Roman Catholic and members of the Revival Centre (n = 4, each). Reflective of the poor knowledge of HPV in Papua New Guinea generally [42], only three women who we interviewed and tested positive for HPV had ever heard of the virus. Ten women had previously had a Pap test and of these only two had ever received their Pap test results, more than three months after their Pap test. Of the 26 women interviewed, none had undergone their three-month clinical review. See Table 1 for more demographic details.

**Table 1:** Participants’ demographic data

Demographics		N=	%
Age range	30–39	12	46
	40–49	10	38
	50+	4	15
Marital Status	Single	1	4
	In a relationship/ Married	19	73
	Separated/ Widowed	6	23
Number of Children	0	3	12
	1–2	9	34
	3+	14	54
Employment Status	Yes	15	58
	No	4	15
	N/A	7	27
Education	Bachelor	3	12
	Diploma/ Certificate	8	30
	Up to Grade 12	13	50
	No school	2	8
Religion	Baptist	2	8
	Catholic	8	31
	Lutheran	5	19
	Not Answered	5	19
	Pentecostal	1	4
	Revival Center	4	15
	Seventh Day Adventist	1	4
Province of Residence	Chimbu	1	4
	Madang	13	50
	Southern Highlands	2	8
	Western Highlands	10	38
Received treatment	No	1	4
	Yes	25	96
Previous Pap test	No (never been screened)	16	62
	Yes	10	38

### Findings

Based on the interpretative phenomenological analysis, three superordinate themes emerged: (1) facing and alleviating initial worries, (2) transforming the disclosure process, and (3) connecting to their faith.

### Facing and alleviating initial worries

Women’s experiences of receiving a positive test result were initially received with mixed feelings. Prior to undertaking HPV testing, all women received a (10–15 min) health education session with information about the virus and its causative association to cervical cancer. In the absence of a national or provincial HPV messaging campaigns, having only heard of the virus and its relationship to cervical cancer for the first time in the health education session prior to testing, most of the women spoke of feeling uncertain of the meaning of their positive test result. The confusion lied in their lack of biomedical knowledge and their attempt to make sense of the new information in a very short period of time. This was complicated further by hearing what HPV type they had, leaving women to wonder what the impact of one strain over the other was. As 38-year-old Rina from Madang stated ‘my results… there are two stages, they said I had 16, and 18, I think. So, what is the difference? Are these categories? Category 16 and 18? I do not understand what that is.’ Similarly, Phillipa, a 47-year-old mother of two, was diagnosed with both HPV 18 and HPV 45 and received same day treatment. When discussing her test result, she was similarly confused by the strains: ‘18 and 45? That’s the zone on the cervix, no? I do not know.’

Belina, a 39-year-old mother of three, sought testing due to a family history of cervical cancer. Her familial background had shaped her own biomedical understanding of what causes the disease (i.e., heredity) and led her to believe that she was at high risk for cancer, yet unclear about how this was manifesting in her body. After being tested and treated in Madang, Belina explained:

I knew all along that it would be positive… it’s something that has been bothering me. I was worried if it’s positive, what will they say, what stage am I in? Am I in the stage of cancer or do I only have the virus but not yet cancer? So, these questions, the thoughts are bothering me… I want to know, what stage I am in now, what is my stage?... but they just said you have the virus. What does it mean?

In the hopes of having clarity on her status, Belina mentioned that she would like to see what the actual impact on her body was and enquired about the possibility of seeing a picture of her cervix. She was unsure whether being able to ‘see’ the impact of the virus on her cervix would alleviate or, instead, heighten her fears of what was biologically happening inside of her. Although she was told by the health care worker that the option of taking a picture of her cervix was not feasible, Belina still wondered whether a picture of her cervix would help her answer any lingering question.

In the absence of symptoms, the ambiguous (and invisible) nature of the impact of the virus on their bodies led many women to feel upset and scared about testing positive for HPV. For twelve of the women who had experienced pain or abnormal symptoms prior to being tested, their positive test result was less surprising, but equally frightening. Women feared for their future and the potential impact it would have on their family and children. Bettina, a single mother of five from Mount Hagen had previously suffered from gynaecological issues. Because of her symptoms, she had decided to undergo a Pap test a few years earlier when the Meripath program was still in operation but had never received her results. Speaking of her experience with HPV S&T, Bettina elaborated about the time she received her positive HPV test result, emotionally sharing:

I just went in, and they tested me, and it was positive. I thought to myself ‘How long will I be with my family, with my kids?’… I don’t want to leave my children because all along I’ve been supporting them alone, as a mother and father… being both, that’s why the recent test result is something that bothered me… I was scared.

Bettina’s positive test result led to feelings of worry about her life and her children’s future. She recognized the impact her test result has on her own temporality of survival and the possibility that, without treatment, her children could be orphaned.

Yet, when recalling the access to same day treatment, these initial worries and fears dissipated. Immediate access to precancer treatment, if eligible, alleviated the uncertainty and negative feelings women experienced when initially given their results. Betty, a 40-year-old married mother of five from Mount Hagen, explained how same day treatment transformed her initial feelings of testing positive.

Well, my test result was positive… 18 or 33 or something? I was like ‘oh what’s 18? What’s 33? Is it going to lead to something else?’ I had that scary thought but it’s like they [health care workers] said, ‘It has been treated [with thermal ablation]’, yeah. So, they got it. They have taken care of it [precancerous lesions].

Rejoya, a 31-year-old mother of an 8-year-old girl, had undergone a Pap test at a private clinic in Madang the previous year. Her sample was sent to Australia for cytology. It took six months for Rejoya to receive her results, at which time she was informed of the presence of abnormal cells on the surface of her cervix. At the clinic she had attended, there were no treatment options available, nor was she recommended any follow up. Referring to her abnormal Pap test result, Rejoya was still anxious about having this ‘woman’s disease’. She was informed of HPV S&T by one of her peers, after having attended the provincial hospital’s gynaecology clinic. Unlike her previous experience, here at the Well Women’s Clinic she was eligible for, and, received thermal ablation, providing relief and reassurance.

At this clinic, the health care worker told me what I was supposed to do, and what the medicine was supposed to do, so they put it in, applied the treatment [thermal ablation]. They told me after six weeks to come back and check for my result. They gave me instructions to follow, I followed every instruction. After six weeks, I came again, they checked me, and I was ok. They said those signs [precancerous lesions] that they saw earlier were not there anymore. Everything went back to normal. I am so happy about that. Like when I came, they were so helpful, the staff. They encouraged me, saying ‘Don’t worry, the treatment that we have applied will make everything alright’. My cervix is back to normal.

Elsa’s experience, a 43-year-old mother of three from Mount Hagen, echoed the overall sentiment of many of the women:

First, I was scared, you know, but my experience with the treatment was positive. It turned out well. Thank goodness! But this is really something … same place, same day you get all your results… same day and you get your treatment. It’s something that, you know, needs to be everywhere… I think all women deserve a service like that.

### Transforming the experience of disclosure

Most women acknowledged that they were initially apprehensive about disclosing their test result to their partners and family members. Much of the apprehension was due to the stigma associated with the STI test result and the associated reactions from family and friends. However, this apprehension dissipated because within a few hours of testing the women had been treated with thermal ablation, made healthy again, by the time they returned home.

Amy, a 34-year-old married mother of one, had heard about HPV S&T on the radio. She had been experiencing pain during intercourse with her husband and thought that she should attend the screen-and-treat program to see if they could help. When she tested positive for HPV and was treated, she contemplated about how best to inform her husband about her screen-and-treat experience:

At first, I didn’t tell my husband that I had been there. I was a little scared. When he came back home [from work], and I told him that I went there, and this was what happened like this, he was very quiet. My husband is always quiet, he just sits down, and he never says anything, he just sits and looks. But this time, he said ‘Sorry this is happening to you. But now we know what is happening, you were treated, and that’s good’. He was very happy and said ‘When [is] your review date?’ and I told him ‘18th July’. He said, ‘Please keep in touch with the sisters [health care worker],’ and he advised me that he will come with me. I am happy I told my husband. We both are very happy.

Amy’s husband instantly became a significant source of support for her, even offering to accompany her for her review appointment.

Belina experienced a similar level of support from her partner. At the time she was living with her father, a pastor, while her partner of a few years was working elsewhere in Papua New Guinea. Unable to tell her father, Belina first told her partner, who helped bridged the communication with her father.

I told him [my partner] right after the test, I cried … I cried and he said ‘Do not worry, you have me now. I’m right behind you, I’ll support you. But do not worry.’ It made me feel good. My partner is the one who told my daddy. He told him everything... My daddy is a man of God. He said, ‘Don’t worry, we’ll pray over it’. Because we are separated [by distance], my partner told my daddy, ‘I will be away so you will be with her always,, please make her happy.’

In the case of same day treatment, the issue of disclosure aided with other aspects of the screen-and-treat experience. It also helped women navigate the ‘after-care treatment’ instructions. Following thermal ablation, women were instructed to abstain from vaginal intercourse for six weeks to allow the healing process to take place. Women in long-term relationships were initially apprehensive that this requirement might create tension in their relationships. This was the case for Kara. At the time of her appointment, her husband of several years was working outside of the main city, and she had expressed some relief knowing she would not have to disclose the six-week abstinence requirement. Kara explained that ‘they [health care workers] told me to abstain from sex for six weeks and I’m glad that my husband is working outside’. When she later decided to disclose her experience to her husband, he was supportive of her experience and needs:

Oh, he was happy that I went for the test and was treated. He said ‘well you got your treatment and, then you will go back for your review again in August’…. I have to go back for another [clinical] review. But he was happy that I went for the test and was treated.

Most women also saw disclosure as an opportunity to advocate for the screen-and-treat service. They felt compelled to inform the women in their lives (i.e., mothers, sisters, and peers) to ensure they get promptly screened. Rima, a 40-year-old married mother of three from Madang explained:

After having gone through this, I called all my sisters [sisters and peers] and told them that they should go and get checked. I said, ‘You all should go and do this check-up’. We kind of joked around at first but then I seriously urged that they should. I have gone there already, and I want you all my sisters to go too. I told them to go make an appointment and then go on the date given and wait to be seen because it is for your own good. So, I told my sisters, my family members and even those women from the street, quite a number of women.

Liana from Madang had a similar purpose for disclosing her test result to close family and friends, noting that:

All my family members know about it [my test result] right now. I’m not in the village but when I get there, I would actually explain to those mothers, trying to tell people [about the service]. Not trying but I will tell those people in the village. Educate them on what is happening to women out there, and I will tell them or advise them to come for that medical check.

Of the women who tested positive for HPV, Nayla, a 34-year-old married mother of two, was ineligible for same day treatment. Upon visual inspection she was informed that she was given a referral to the Gynaecologist for further examination. Clearly distraught of learning that she was positive for HPV and needing a specialist referral, Nyla cried with her husband when he came to pick her up from the clinic.

I was in tears telling him about my result, he was there to comfort me, encourage me so he told me ‘It’s not confirmed that you have cancer, it’s a virus, there’s always hope’. I felt better. And when I spoke to Tambu [mother-in-law], she told me not to worry, she said I’ve been there for a check-up and … so she was encouraging. It helped me.

### Connecting to their faith

Religiosity played an important role in women’s experience of testing positive for HPV and receiving treatment on the same day. For them, access to treatment was considered a divine intervention. Elsa, a 43-year-old mother of two from Madang, reflected after her treatment, that ‘I believe in God, so I felt blessed that I was healed by God, and I was guided by the Lord all through my operation [procedure], before and after, meaning I didn’t feel any pain.’ Just like Elsa, Amy, a 34-year-old mother of one, was convinced that prayer is what helped her. She explained that when she was diagnosed with HPV, ‘I prayed to God ‘please just remove all this and just give me mercy and peace’. And I received this treatment. It was God.’

Kiana, a 34-year-old mother of four from Mount Hagen, described how God healed the women:

God is the only one that holds our lives. God brings the doctors and the medicine. Whether this cervical cancer develops from food, from our own mistakes as women like not being faithful to our husbands, or the husbands not being faithful to us, the thing is, we must be with God, that is the important thing. God holds our lives. And now I’m fine.

Kiana’s statement reveals that she believes in God being the source for all events happening, whether good or bad. To her, God is the source and the answer.

Having a strong sense of religious faith helped the women remain positive about their overall experience and their future.

## Discussion

A same day HPV-based screen-and-treat clinical algorithm is distinct from traditional screening programs, in that it provides access to same day results and treatment for eligible women who test positive for oncogenic HPV types. The World Health Organization (WHO) only recommended HPV screen-and-treat programs in 2018, so the social research that examines women’s experiences of undergoing a same day HPV-based screen-and-treat service is greatly lacking, with no such studies published to date, highlighting the importance of this research.

In contrast to other qualitative research on HPV, these women were provided with same day treatment, enabling them to achieve quicker resolution about the ongoing social as well as medical meaning of their diagnosis. Rather than going home dreading their risk of cervical cancer from being diagnosed with the cancer causing STI, and being “marked”, they returned home having had the possible existential threat resolved, with no further action required than abstain from sexual intercourse for 6 weeks and to have a follow up in 5 years’ time.

Disclosure was found to be a critical finding in the women’s experiences. All women in the study, including Nayla who was ineligible for treatment, spoke positively about disclosing their participation in the service and their HPV status to their partners. This is not the case in other studies where being diagnosed with HPV and disclosing the diagnosis of this STI brings a negative response from others. Previous research focusing on disclosure found similar concerns including fear of being stigmatized by family and friends by having to disclose a positive STI test result, more so due to the lack of available treatment or follow-up [32, 33]. In the case of HPV, a systematic review [54] has shown that women feared the negative (i.e., promiscuous) shift of their social identity due to the stigma and shame associated with an untreated positive HPV test result. In some of these studies, women were screened using a pap smear, which does not detect oncogenic HPV types, but ‘labels’ the women as having abnormal cells (or abnormal results which indicate cervical cell changes). Further, none of these programs offered same day treatment. Compared to previous cervical screening programs which lacked access to immediate treatment (i.e., immediate resolution), it seems that, with HPV S&T, women’s positive experience with disclosure is associated with having had the threat of cervical cancer resolved on the same day with the screen and treat intervention. Until this same day test and treat program is scaled up and evaluated elsewhere, it is unclear if these narratives are an anomaly or not. Ongoing qualitative research into disclosure is warranted, including ensuring that the experiences of women who report adverse responses to their disclosure are captured. Further research would enrich the literature to capture both positive and negative experiences of disclosure and examine women’s needs during the disclosure process (i.e., messages, communication methods, support during disclosure).

Religiosity also played an important role in the women’s experiences. Women viewed the biomedical technology (i.e., thermal ablation) as a divine intervention, made only possibly by God. Previous research has asserted that religiosity and faith are significant elements of the health care experience in Papua New Guinea [43–46]. This is also inherently linked to the concept of morality, an important aspect of Papua New Guinea’s moral framework in understanding illness and biomedicine [44], which was found to be an important finding in our study.

In reflection, these initial findings lead to wider discussions on the potential impact of HPV S&T on HPV stigma. Most of the HPV stigma and access to screening literature shows that lack of knowledge around HPV, ‘stigma marking’ (i.e., HPV being linked to immoral sexual behaviours), and the association with fatality increase the experience of internalized stigma for women who test positive for HPV [47]. Furthermore, women’s prolonged HPV status (without resolution) due to the lack of access to treatment exacerbates these stigma experiences. Yet, this study begs to ask the question ‘can access to same day screen-and-treat reduce stigma associated with treatable HPV diagnoses?’

The literature on another well-known stigmatized STI, HIV, offers an opportunity to dive into the validity of this assumption. Some studies have shown that access to anti-retroviral (ART) has beneficial impacts on overall well-being and quality of life [48] and can help reduce HIV-related stigma by addressing common misperceptions [48, 49]. Some studies go as far as to suggest that access to ART normalizes HIV, making it more manageable [50, 51]. The positive impact of access to ART on stigma can potentially be attributed to comprehensive HIV testing services (HTS), including wider reach health education of HIV, increasing knowledge about HIV transmission modes and prevention practices, and dispelling any stigma-inducing misconceptions [49].

Certainly, our study has proven the beneficial impacts on women who test positive with HPV overall well-being. Yet, due to the novel clinical algorithm and the lack of social research evidence on this issue, it is premature, at this time, to make such a confirmation statement. Further research into the topic should be undertaken to confirm the distinct link between access to same day HPV screen-and-treat and reduced HPV stigma.

### Strengths and limitations

This is the first study of its kind in Papua New Guinea, and, to our knowledge, globally. This study could serve as a blueprint for similar qualitative studies looking at same day HPV-based screen-and-treat programs in similar settings. Using a qualitative approach, particularly the use of interpretative phenomenological analysis helped emphasize women’s stories which allowed for a deeper inquiry into their experiences. Nevertheless, there are some limitations to the study. Twenty-six is a relatively adequate sample in qualitative research. Yet, interviewing more women who tested positive for HPV and treated on the same day could have helped to substantiate the findings and/or yield additional findings that were not discussed in this paper. In addition, stories and experiences of women who were not eligible for treatment is extremely critical. Amplifying the voices of women ineligible for precancer treatment would highlight the need to make cervical cancer treatment services available and accessible to any women at risk in the country, as well as improve social and health care services for all women in need. Another distinct limitation is the consistent positive experience with disclosure. It is imperative to capture all experiences associated with disclosure to better understand women’s needs with the process of divulging sensitive experiences. This limitation also prompts the need for additional data collection methods (i.e., surveys, questionnaires) that would allow the participants to speak to their experience of disclosure anonymously and candidly. Further, the study only interviewed a few of the women who were back at the WWC for their (3-month) clinical review. Additional follow-up is needed with women after clinical review to ensure we add to the evidence base of women’s entire experience post-screen-and-treat.

## Conclusion

As the world moves towards the implementation of HPV screen-and-treat, this study hopes to shed light on the meaning and experiences of a positive HPV test result and how women navigate the impact of this test result and subsequent treatment. This study has highlighted key elements of navigating this new clinical algorithm. It found that although women experienced initial uncertainties pertaining to their positive HPV test result, which was associated to the lack of understanding around the test result and its implications, undergoing same day treatment resolved the women’s concerns, making this program highly acceptable and radically changing their illness narrative. Same day HPV screen-and-treat transformed women’s experiences of testing positive and left them feeling hopeful about their future, and the future of all Papua New Guinea women.

## Data Availability

The data used and analyzed for this study were not made publicly available to protect participants’ privacy but are available from the corresponding author on reasonable request.

## Acronyms

HPV: Human Papillomavirus
HPV S&T: single visit HPV-based self-collect, test, and treat screening strategy
IMR: Institute of Medical Research
IRB: Institutional Review Board
MRAC: Medical Research Advisory Committee
NDoH: National Department of Health
NHMRC: National Health and Medical Research Council
PAP: Papanicolaou smear
Papua New Guinea: Papua New Guinea
PNGIMR: Papua New Guinea Institute of Medical Research
STI: Sexually Transmitted Infections
UNSW: University of New South Wales
WHO: World Health Organization
WWC: Well Women’s Clinic
